# The Potential Influence of Common Viral Infections Diagnosed during Hospitalization among Critically Ill Patients in the United States

**DOI:** 10.1371/journal.pone.0018890

**Published:** 2011-04-29

**Authors:** Makesha Miggins, Anjum Hasan, Samuel Hohmann, Frederick Southwick, George Casella, Denise Schain, Huazhi Liu, Azra Bihorac, Lyle Moldawer, Philip Efron, Darwin Ang

**Affiliations:** 1 Department of Surgery, University of Florida, Gainesville, Florida, United States of America; 2 Department of Infectious Disease, University of Florida, Gainesville, Florida, United States of America; 3 University HealthSystem Consortium, Gainesville, Florida, United States of America; 4 Department of Statistics, University of Florida, Gainesville, Florida, United States of America; 5 Department of Anesthesiology, University of Florida, Gainesville, Florida, United States of America; 6 Critical Care, University of Florida, Gainesville, Florida, United States of America; The University of Hong Kong, Hong Kong

## Abstract

Viruses are the most common source of infection among immunocompetent individuals, yet they are not considered a clinically meaningful risk factor among the critically ill. This work examines the association of viral infections diagnosed during the hospital stay or not documented as present on admission to the outcomes of ICU patients with no evidence of immunosuppression on admission. This is a population-based retrospective cohort study of University HealthSystem Consortium (UHC) academic centers in the U.S. from the years 2006 to 2009. The UHC is an alliance of over 90% of the non-profit academic medical centers in the U.S. A total of 209,695 critically ill patients were used in this analysis. Eight hospital complications were examined. Patients were grouped into four cohorts: absence of infection, bacterial infection only, viral infection only, and bacterial and viral infection during same hospital admission. Viral infections diagnosed during hospitalization significantly increased the risk of all complications. There was also a seasonal pattern for viral infections. Specific viruses associated with poor outcomes included influenza, RSV, CMV, and HSV. Patients who had both viral and bacterial infections during the same hospitalization had the greatest risk of mortality RR 6.58, 95% CI (5.47, 7.91); multi-organ failure RR 8.25, 95% CI (7.50, 9.07); and septic shock RR 271.2, 95% CI (188.0, 391.3). Viral infections may play a significant yet unrecognized role in the outcomes of ICU patients. They may serve as biological markers or play an active role in the development of certain adverse complications by interacting with coincident bacterial infection.

## Introduction

Viruses are ubiquitous in the environment and are the most common source of infection among immunocompetent individuals. Despite their pervasiveness, viral infections are generally not considered to be of clinical significance among the critically ill, unless the patient is significantly immuncompromised. Much is known about the progression of viral infections as opportunistic pathogens among immunocompromised patients while little is known about their role in patients who are immunocompetent before hospitalization. Even among patients with no history of immunosuppression, reactivation of viral herpes viruses (cytomegalovirus (CMV) and herpes simplex virus (HSV)) has been documented in critically ill [1]–[10]. This suggests that at least for some critically ill patients with no pre-hospital diagnosis of immunosuppression, newly acquired or reactivation of viral infections during hospitalization can be clinically significant in this patient population.

Using a large population based database, we sought to examine the association of viral infections to the outcomes of ICU patients with no evidence of infection or immunosuppression on admission, and to identify potential variables amenable to intervention. We hypothesize that acquired viral infections during hospitalization may carry their own risk for adverse outcomes among patients in the intensive care unit.

## Methods

### Data Source and Study Population

A population based retrospective cohort study using the University HealthSystem Consortium (UHC) Database, specifically searching the Resource Manager and Clinical Database within the UHC system, was conducted. The UHC is comprised of 103 academic institutions and 210 affiliate institutions representing over 90% of all academic medical centers in the United States. The UHC database is an administrative database, comprised of International Classification of Disease-9 (ICD-9) diagnosis and procedure codes. At present, it is the only population based dataset to contain information on the critically ill and their exposure to viral and bacterial infections.

Only patients without a diagnosis of infection present on admission were included in the study. The inclusion criteria included immunocompetent ICU patients greater than or equal to eighteen years of age admitted to the hospital between the third quarter, 2006, and second quarter, 2009. Patients with a primary or secondary ICD 9 code signifying a history of organ transplantation, human immunodeficiency virus (HIV) or acquired immunodeficiency syndrome (AIDS), autoimmune disease, leukemia, pancytopenia, or lymphoma were excluded. Immunocompetency was defined as the absence of the aforementioned diagnoses. A total 209,695 patients met the study criteria.

### Study Cohorts and Outcomes

ICD-9 codes present at hospital discharge were used to divide patients into four cohorts: (1) individuals without a documented infection (negative); (2) those who were diagnosed with a viral infection during their hospitalization (viral); (3) those with a documented bacterial infection (bacterial) during their hospitalization; and, (4) those who were diagnosed with both bacterial and viral infections during the same hospital admission (coincident). Although the dataset does not allow us to determine if the infections overlapped or occurred simultaneously, we referred to this cohort as “coincident” for simplification. The reference group for all relative risk calculations was the group which did not have a documented infection. For pneumonia, sepsis, and septic shock, diagnoses that by definition require the presence of an infection for diagnosis, a subset analysis was also performed using the bacterial cohort as the reference group. Six hundred ninety-eight documented viral infections were identified. Eight outcomes frequently encountered in ICU patients were analyzed; death, pneumonia, acute respiratory distress syndrome (ARDS), respiratory failure, diarrhea, multi-system organ failure (MSOF), sepsis, and septic shock.

### Analysis

Statistical analysis was performed with SAS (version 9.2; SAS Inc., Cary, NC). Parametric data expressed as proportions were evaluated by Chi Square tests, while nonparametric data were evaluated by Fisher's exact test. ANOVA was used to calculate p-values for multiple proportions. Multivariate analysis by Poisson regression was used to calculate the univariate and multivariate adjusted relative risks of each exposure group. Variables were identified as potential confounders if it could be rationalized that an independent effect on both the exposure and outcome occurred without influencing the pathway of interest. The final multivariate regression included age, gender, race, and hospital cluster. Adjustment for hospital cluster accounts for patient volume and prevents over or under representation by hospital size. Seasonal distribution of infections was determined using the hospital admission date. The Cochran-Armitage test was used to test for trend. ICD 9 codes for common respiratory and gastrointestinal tract viruses were used to determine which organisms alone or in combination with a bacterial infection are associated with the outcomes of interest.

Measures of potential biological interaction between bacterial and viral coincident infections were estimated by deviation from additivity methods described by Rothman and others [11]–[13]. This method requires the calculation of the RERI (relative risk due to interaction), AP (attributable risk proportion due to interaction), and S (the synergy index). No biological interaction is present when the RERI and AP are equal to zero and synergy index is equal to one.

## Results

**Figure 1:**
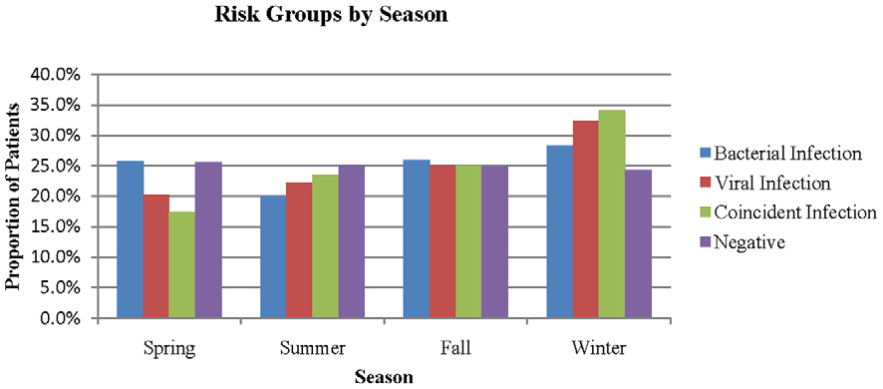
Proportion of individual risk groups by season.

**Figure 2:**
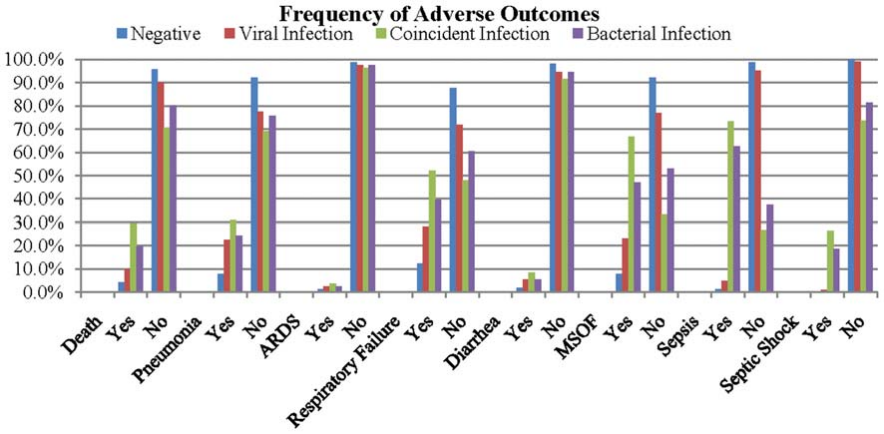
Frequency of adverse outcomes.

Significant differences did exist among socio-demographic characteristics of the cohorts, most notably age and average hospital length of stay (Table 1). There was seasonal variation for viral infections. The seasonal distribution of infections for each cohort increased from spring to winter, however only the viral and coincident cohorts exhibited a linear pattern (Figure 1). There were significantly more documented bacterial infections compared to both viral and coincident infections. Comparing disease proportions, the coincident cohort tended to have higher frequencies for each adverse outcome (Figure 2). Notably, length of stay was also highest in the coincident group. The average length of stay was 11.5 days for the negative cohort, 21 days for the virus cohort, 32 days for the bacteria group, and 46.2 days for the coincident cohort (p-value <0.05).

**Table 1:** Risk group sociodemographics.

	Negative	Viral Infection	Coincident Infection	Bacterial Infection	p-values
	N = 190,386	N = 370	N = 328	N = 18, 611	
Gender					
Male	53.2%	52.4%	53.1%	53.9%	0.28
Female	46.8%	47.6%	46.9%	46.1%	0.28
Race					
White	68.8%	66.9%	65.1%	65.7%	<0.0001
Black	15.3%	16.3%	15.3%	17.9%	<0.0001
Hispanic	7.0%	8.9%	7.3%	6.6%	0.19
Asian	2.4%	2.7%	5.5%	2.8%	<0.0001
Others	6.6%	5.2%	6.7%	6.9%	0.16
Age (yrs)					
18∼20	3.0%	1.1%	2.4%	2.1%	<0.0001
21∼30	9.7%	7.6%	4.3%	6.7%	<0.0001
31∼40	10.4%	10.0%	7.9%	7.8%	<0.0001
41∼50	15.9%	17.3%	17.1%	13.5%	<0.0001
51∼60	20.3%	29.5%	27.1%	20.6%	<0.0001
61∼70	19.0%	18.9%	22.6%	20.9%	<0.0001
71∼80	14.3%	10.3%	12.5%	17.7%	<0.0001
>80	7.4%	5.4%	6.1%	10.6%	<0.0001
Mean Age	54.8	54.8	56.7	58.7	
Median Age	56	55	58	60	
Maximum Age	108	91	96	107	
Minimum Age	18	18	19	18	
Insurance Status					
Commercial/Private	37.4%	33.8%	32.0%	27.1%	<0.0001
Medicaid	11.5%	19.2%	18.3%	14.2%	<0.0001
Medicare	35.9%	34.1%	36.6%	45.4%	<0.0001
State Assisted Healthcare	1.2%	1.4%	1.5%	1.0%	0.02
Military	1.4%	0.8%	1.5%	1.2%	0.13
Auto Insurance	2.2%	1.4%	0.9%	2.2%	0.25
Workers Compensation	1.6%	1.4%	2.1%	1.2%	0.0008
Research	0.03%	0%	0%	0.03%	0.97
Title V Maternal & Child Health	0.03%	0%	0%	0.06%	0.37
County Medically Indigent Services	1.0%	0.8%	1.2%	0.9%	0.62
Charity	1.0%	1.6%	0.9%	0.9%	0.27
Self-Pay	5.1%	3.5%	3.7%	3.9%	<0.0001
Other NOS	1.0%	1.4%	0.9%	1.2%	0.004
Unknown	0.7%	0.8%	0.3%	0.7%	0.62

The relative risk (RR) for each outcome according to risk group is shown in Table S1. Viral, bacterial, and coincident cohorts were at risk for all adverse events compared to the reference groups. Coincident infections had the strongest associations with adverse events, especially for mortality RR 6.58 95% CI (5.47, 7.91); MSOF RR 8.25 95% CI (7.50, 9.07); and septic shock RR 271.2 95% CI (188.0, 391.3) when negative was used as the reference group. For these three adverse events, the confidence intervals among the coincident cohort were beyond the confidence intervals of either the viral or bacterial only cohorts. When bacterial infection was used as the reference group, viral infections were associated with an increased risk of pneumonia RR 1.30 95% CI (1.10, 1.55), sepsis RR 1.18 95% CI (1.10, 1.28), and septic shock RR 1.48 95% CI (1.23, 1.78).

Table S2 lists the relative risks for each outcome according to infecting organism. An increased risk of death was associated with several viral species including coronavirus, CMV, HSV, influenza virus, and respiratory syncytial virus (RSV). The combination of influenza with a bacterial infection was associated with pneumonia RR 4.65, 95% CI (3.22, 6.70), respiratory failure RR 4.21, 95% CI (2.39, 7.40), diarrhea RR 4.38 95% CI (1.28, 14.98), MSOF RR 5.17, 95% CI (3.38, 7.91) sepsis RR 45.1, 95% CI (28.1, 72.6), and septic shock RR 122.2, 95% CI (33.9, 440.2). Influenza alone was only associated with an increased risk of respiratory failure 3.19, 95% CI (1.96, 5.17). CMV alone RR 4.69, 95% CI (2.95, 6.28) and with bacterial infection RR 4.30, 95% CI (2.95, 6.28) were associated with an increased risk of pneumonia. HSV with bacterial infection (RR 4.28 95% CI 3.23, 5.67) and alone (RR 3.60 95% CI2.68, 4.85) were associated with an increased risk of pneumonia. Adenovirus and coronavirus with bacterial infection as well as RSV alone were also associated with pneumonia.

MSOF was associated with the presence of CMV RR 4.79, 95% CI (8.64, 11.90) as well as HSV RR 2.37, 95% CI (1.62, 3.46). The presence of CMV, HSV, and influenza virus with bacterial infection was associated with an increased risk of septic shock; RR 218.7 95% CI (128.1, 373.4), 309.2 (211.6, 452.0), 122.2 (33.9, 440.2), respectively. HSV in the absence of bacterial infection was also associated with septic shock, RR 33.71, 95% CI (11.78, 96.41).

There was significant biological interaction between viral and bacterial infections for septic shock, MSOF, and death. For septic shock, the risk excess, or RERI, due to the interaction between viral and bacterial infection was 127.7 95% CI (32.8, 222.5); the attributable proportion or AP was 0.23 95% CI (0.27, 0.51); and the synergy index was significant at 1.5 95% CI (1.2, 1.9). For MSOF, the RERI was significant at 10.6 95% CI (5.1, 15.9); AP was 0.43 95% CI (0.32, 0.58); and the synergy index was significant at 1.9 95% CI (1.5, 2.4). Although the RERI was not significant for death, the AP and synergy index were significant at 0.28 95% CI (0.046, 0.45) and 1.39 95% CI (1.02, 1.88) respectively.

## Discussion

Viral infections diagnosed after hospital admission may play a significant yet unrecognized role in the outcomes of ICU patients. While they are the most common organism of infection in immunocompetent adults, they have not been considered to be a serious risk factor for those critically ill patients who are admitted to the ICU without a diagnosis of infection or known immunosuppression. Few studies have examined the role of specific viral infections among patients who are immunocompetent before admission into the ICU despite the fact that there is no data to support that these patients are less likely to develop viral infections in this setting compared to their immunocompromised counterparts. Thus the implications of any positive findings may have the potential to affect all ICU patients.

The results of this population based study show that when a viral infection is present either alone or during the same hospitalization as a bacterial infection, it is associated with an increased risk of mortality, pneumonia, ARDS, respiratory failure, diarrhea, MSOF, sepsis, and septic shock. Perhaps the most significant finding is that coincident infections show the strongest association with each adverse outcome. This is particularly true for death, septic shock and MSOF where the test for synergistic interaction is significant. In addition for MSOF and death, the 95% confidence interval is outside the bounds of the other risk groups. These findings suggest that the coincident group carries its own independent association. In the subset analysis, viral specific associations were also found to be significant. For viruses that are latent and are known to reactivate, CMV and HSV were significantly associated with mortality, pneumonia, ARDS, respiratory failure, diarrhea, MSOF, sepsis, and septic shock. Patients with viruses that have an active phenotype such as adenovirus, coronavirus, RSV, and influenza virus had a significant risk of death, pneumonia, respiratory failure, MSOF, sepsis and septic shock.

If the association between viral infections and immunocompetent ICU patients represents a true clinical relationship, then these findings have several potential implications. These implications range from viral infections as markers for disease to viral infections participating in the causal pathway of complications such as ARDS and MSOF as well as the occurrence of other infections. Some of our findings have already been confirmed in recent literature, such as the link between CMV and HSV with mortality, pulmonary disease, sepsis, and overall poor patient outcomes [1]–[3], [5]–[10], [14]–[19]. Studies have reported reactivation of CMV and HSV in up to 35% and 64% of critically ill patients, respectively [8]–[10], [16], [17], [20]. In a study involving patients with ventilator associated pneumonia, Luyt et al reported 21% of patients with HSV isolated from bronchoalveolar lavage (BAL) specimens had HSV bronchopneumonitis [8]. It is reasonable to assume that a significant proportion of critically ill patients are at risk for reactivation of these herpes viruses. Between 50% and 80% of the population is CMV-seropositive by age 40 years and 72% of the population aged 14–19 years is seropositive for HSV-1 and HSV-2 combined. [6], [9], [18], [19], [21]–[23] In our study, HSV was associated with an increased risk of each outcome while CMV was not associated with sepsis or septic shock in patients without an additional documented bacterial infection during their hospitalization.

Some have suggested that viral detection does not equate to viral disease. However, it may serve as a marker for increased risk of disease development. van den Brink et al addressed the concept of viral reactivation as a marker as opposed to a mediator of pulmonary disease in patients with HSV-1 isolated from bronchoalveolar lavage (BAL) samples [21]. The results of our study also suggest that there is an independent association of death and MSOF among patients who develop both a viral and bacterial infection during their hospitalization. MSOF has been attributed to septic shock secondary to bacteremia, but there are patients who may not develop MSOF despite the presence of bacteremia.

The relationship of viral infections to outcomes in the ICU is more complicated than the documentation of viral infection. The mechanism and extent to which viruses cause disease is diverse. Viruses belonging to the Herpesviridae family are known for their ability to become dormant in host cells while other viruses such as influenza virus, adenovirus, RSV, and coronavirus are only capable of causing disease at the time of infection. RSV, the only non herpes virus in our study associated with death in the absence of bacterial infection, carried the highest risk of mortality. RSV is a common community pathogen making it an unlikely nosocomial infection unless the patients were mechanically ventilated or had a nasogastric tube in place. Rouby et al demonstrated an association between oral and nasal intubation with the development of sinusitis which facilitates respiratory infections in the ICU [24].

The concept of bacterial superinfection is common in viral respiratory illnesses [25]. Epidemiological evidence of synergistic relationships between viruses and bacteria can be seen in influenza pandemics where an increased incidence of *Staphylococcus aureus* pneumonia was encountered as well as an increased incidence of bacterial otitis media associated with both RSV and influenza virus [26]. Viral infections may facilitate bacterial colonization by damaging host respiratory epithelium, making bacterial adherence or invasion easier. Preceding or intercurrent viral infections may down regulate the host immune response leading to subsequent bacterial infection [25], [26]. It is therefore possible that treatment of viral infection or viral reactivation in this setting may mitigate against the association with poor outcomes seen in coincident infection.

The limitations inherent in this study are the degree of bias, the inability to infer causality, and classification of critically ill patients as immunocompetent. While the purpose of this study was to identify associations, it is also meant to promote hypothesis generation to encourage prospective studies that will address causality. We defined immunocompetent as the absence of several diagnoses associated with immunosuppression. While classifying patients in the ICU as having acquired immunosuppression due to prolonged critical illness appears to be a valid assumption, this is supported by limited rigorous scientific data. Therefore the premise remains controversial. Although studies have demonstrated alterations in immune system function in the critically ill, this may represent a transition along the spectrum of immunocompetency opposed to true immunosuppression. Another limitation found in administrative databases is the lack of clinical data. Key variables are missing to ascertain the site from which the virus was isolated or the order of clinical events. We do know these infections occurred within the same hospitalization, were not documented as present on admission, and were significantly associated with worse patient outcomes. Considering viral infections are rarely diagnosed in the ICU, it is possible that only those patients who were most critically ill were tested for viral infections. Thus, the effect we see is one of severity of critical illness instead of viral association. However, patients with only viral infections actually had less risk of adverse outcomes compared to their bacterial and coincident counterparts. This may reject some of this bias. It is likely that viral infections were rarely thought of as the source of poor outcomes among patients whose pre-hospital status was not immunocompromised. Because the denominator of active viral infection among the critically ill is not known, the data presented in this paper may significantly underestimate the impact of viral infections in this setting. Despite many of these limitations, this is the only population based dataset that has documented hospital acquired viral infections among the critically ill.

In conclusion, viral infections may play a very important role in the overall outcome of critically ill patients who were immunocompetent before hospital admission. Viral infections may represent an unrecognized health exposure that puts all critically ill patients at risk. They may have potential roles in critical illness as biologic markers or active contributors to specific adverse outcomes. Although no causal relationship can be inferred from these results, the strength of independent association is such that it is reasonable to reconsider the role that viral infections play in the outcomes of immunocompetent ICU patients. Prospective studies should aid in confirming or redefining the role of viral infections among all critically ill patients.

## Supporting Information

Table S1 **Relative risk of adverse outcomes by cohort.**
(DOC)

Table S2 **Significant relative risk of adverse outcome by virus type.**
(DOC)
