# Supplementary material for: The Potential Influence of Common Viral Infections Diagnosed during Hospitalization among Critically Ill Patients in the United States
Source: PLoS One. 2011 Apr 29;6(4):e18890. doi: 10.1371/journal.pone.0018890 (PMC3091021; doi:10.1371/journal.pone.0018890)
Supplement: Table S1 — Relative risk of adverse outcomes by cohort. (DOC) [file pone.0018890.s001.doc]

**Table 2.** Relative risk of adverse outcomes by cohort

| **Outcome** | **Crude Relative Risk** | **Adjusted Relative Risk*** |
| --- | --- | --- |
|  | **RR (95% CI)** | **RR (95% CI)** |
| **Death** |  |  |
| Viral Infection | 2.29 (1.69, 3.11) | 2.36 (1.73, 3.24) |
| Coincident^+^ | 6.70 (5.66, 7.94) | 6.58 (5.47, 7.91) |
| Bacterial Infection | 4.52 (4.36, 4.68) | 4.13 (3.76, 4.53) |
| Negative | Reference | Reference |
| **Pneumonia** |  |  |
| Viral Infection | 2.87 (2.38, 3.48) | 2.84 (2.21, 3.65) |
| Coincident | 3.94 (3.35, 4.64) | 3.91 (3.29, 4.63) |
| Bacterial Infection | 3.10 (3.01, 3.19) | 3.00 (2.82, 3.20) |
| Negative | Reference | Reference |
| Viral Infection | 1.27 (1.08, 1.50) | 1.30 (1.10, 1.55) |
| Coincident | 0.93 (0.77, 1.12) | 0.95 (0.74, 1.21) |
| Bacterial Infection | Reference | Reference |
| **ARDS** |  |  |
| Viral Infection | 1.76 (0.92, 3.36) | 1.79 (0.92, 3.47) |
| Coincident | 2.65 (1.52, 4.62) | 2.64 (1.26, 5.51) |
| Bacterial Infection | 1.86 (1.69, 2.05) | 1.81 (1.55, 2.13) |
| Negative | Reference | Reference |
| **Respiratory Failure** |  |  |
| Viral Infection | 2.28 (1.94, 2.69) | 2.29 (1.84, 2.84) |
| Coincident | 4.24 (3.82, 4.70) | 4.18 (3.68, 4.75) |
| Bacterial Infection | 3.21 (3.14, 3.28) | 3.07 (2.84, 3.32) |
| Negative | Reference | Reference |
| **Diarrhea** |  |  |
| Viral Infection | 2.89 (1.88, 4.42) | 2.89 (1.87, 4.46) |
| Coincident | 4.56 (3.19, 6.50) | 4.47 (3.00, 6.66) |
| Bacterial Infection | 2.94 (2.74, 3.14) | 2.79 (2.47, 3.16) |
| Negative | Reference | Reference |
| **MSOF** |  |  |
| Viral Infection | 2.90 (2.41, 3.50) | 2.92 (2.39, 3.58) |
| Coincident | 8.43 (7.80, 9.12) | 8.25 (7.50, 9.07) |
| Bacterial Infection | 5.94 (5.82, 6.07) | 5.42 (5.06, 5.81) |
| Negative | Reference | Reference |
| **Sepsis** |  |  |
| Viral Infection | 4.02 (2.55, 6.31) | 4.01 (2.41, 6.65) |
| Coincident | 60.4 (55.9, 65.3) | 60.3 (50.5, 71.8) |
| Bacterial Infection | 51.6 (49.5, 53.8) | 50.9 (43.0, 60.2) |
| Negative | Reference | Reference |
| Viral Infection | 1.17 (1.10, 1.25) | 1.18 (1.10, 1.28) |
| Coincident | 0.08 (0.05, 0.12) | 0.08 (0.05, 0.13) |
| Bacterial Infection | Reference | Reference |
| **Septic Shock** |  |  |
| Viral Infection | 11.25 (4.20, 30.13) | 11.26 (4.00, 31.66) |
| Coincident | 275.9 (219.0, 347.7) | 271.2 (188.0, 391.3) |
| Bacterial Infection | 193.3 (166.7, 224.1) | 183.3 (134.2, 250.5) |
| Negative | Reference | Reference |
| Viral Infection | 1.43 (1.19, 1.71) | 1.48 (1.23, 1.78) |
| Coincident | 0.06 (0.02, 0.15) | 0.06 (0.02, 0.16) |
| Bacterial Infection | Reference | Reference |

*Adjusted for age, gender, race, and hospital cluster

^+^Coincident denotes the presence of both bacterial and viral infections
