# Supplementary material for: The Potential Influence of Common Viral Infections Diagnosed during Hospitalization among Critically Ill Patients in the United States
Source: PLoS One. 2011 Apr 29;6(4):e18890. doi: 10.1371/journal.pone.0018890 (PMC3091021; doi:10.1371/journal.pone.0018890)
Supplement: Table S2 — Significant relative risk of adverse outcome by virus type. (DOC) [file pone.0018890.s002.doc]

**Table 3** Significant relative risk of adverse outcome by virus type

| **Outcome** | **Crude Relative Risk** | **Adjusted Relative Risk^*^** |
| --- | --- | --- |
|  | **RR (95% CI)** | **RR (95% CI)** |
| **Death** |  |  |
| Coronavirus Coincident^+^ | 5.73 (1.05, 31.3) | 9.01 (2.47, 32.9) |
| CMV Coincident | 6.61 (4.31, 10.13) | 6.66 (4.35, 10.19) |
| CMV | 4.24 (1.92, 9.36) | 4.21 (2.07, 8.55) |
| HSV Coincident | 5.19 (3.76, 7.15) | 4.92 (3.43, 7.06) |
| HSV | 2.19 (1.30, 3.67) | 2.35 (1.37, 4.05) |
| Influenza Coincident | 2.86 (0.99, 8.25) | 2.75 (1.09, 6.94) |
| RSV | 11.45 (2.86, 45.79) | 11.76 (6.47, 21.39) |
| Bacterial Infection | 4.52 (4.36, 4.68) | 4.12 (3.75, 4.53) |
| **Pneumonia** |  |  |
| Adenovirus Coincident | 12.81 (1.80, 90.95) | 12.91 (11.58, 14.40) |
| Coronavirus Coincident | 3.20 (0.59, 17.5) | 4.78 (1.25, 18.3) |
| CMV Coincident | 4.19 (2.60, 6.74) | 4.30 (2.95, 6.28) |
| CMV | 4.74 (2.55, 8.82) | 4.69 (2.95, 7.44) |
| HSV Coincident | 4.30 (3.19, 5.81) | 4.28 (3.23, 5.67) |
| HSV | 3.49 (2.52, 4.81) | 3.60 (2.68, 4.85) |
| Influenza Coincident | 4.80 (2.50, 9.24) | 4.65 (3.22, 6.70) |
| Influenza | 2.14 (0.69, 6.62) | 2.11 (0.53, 8.45) |
| RSV | 6.41 (0.90, 45.48) | 6.02 (2.00, 18.15) |
| Bacterial Infection | 3.10 (3.01, 3.19) | 2.80 (2.70, 2.90) |
| **ARDS** |  |  |
| CMV Coincident | 4.12 (1.41, 12.01) | 4.17 (1.39, 12.53) |
| CMV | 5.36 (1.41, 20.34) | 5.33 (1.46, 19.42) |
| HSV Coincident | 3.39 (1.55, 7.41) | 3.32 (1.57, 7.00) |
| HSV | 2.66 (1.12, 6.29) | 2.40 (1.00, 5.78) |
| Influenza Coincident | 3.01 (0.44, 20.54) | 3.04 (0.41, 22.49) |
| Influenza | 4.02 (0.60, 27.01) | 4.21 (0.68, 26.13) |
| Bacterial Infection | 1.86 (1.69, 2.05) | 1.81 (1.55, 2.13) |
| **Respiratory Failure** |  |  |
| Adenovirus Coincident | 8.12 (1.14, 57.68) | 9.11 (7.86, 10.57) |
| Coronavirus Coincident | 2.03 (0.37, 11.09) | 3.10 (0.76, 12.70) |
| CMV Coincident | 3.91 (2.64, 5.78) | 4.08 (2.84, 5.86)) |
| CMV | 3.61 (2.05, 6.36) | 3.62 (2.23, 5.86) |
| HSV | 2.45 (1.80, 3.33) | 2.21 (1.62, 3.00) |
| HSV Coincident | 3.94 (3.07, 5.05) | 3.89 (3.20, 4.72) |
| Influenza Coincident | 4.40 (2.56, 7.58) | 4.21 (2.39, 7.40) |
| Influenza | 3.16 (1.51, 6.63) | 3.19 (1.96, 5.17) |
| RSV | 8.12 (2.03, 32.49) | 8.42 (6.02, 11.77) |
| Bacterial Infection | 3.21 (3.14, 3.28) | 2.88 (2.80, 2.96) |
| **Diarrhea** |  |  |
| CMV Coincident | 6.16 (2.90, 13.08) | 5.98 (2.63, 13.63) |
| CMV | 9.88 (4.48, 21.82) | 9.76 (5.07, 18.79) |
| HSV Coincident | 5.00 (2.92, 8.58) | 4.75 (2.81, 8.04) |
| HSV | 3.53 (1.88, 6.65) | 3.55 (1.88, 6.69) |
| Influenza Coincident | 4.45 (1.18, 16.77) | 4.38 (1.28, 14.98) |
| Influenza | 2.97 (0.44, 19.93) | 3.25 (0.48, 21.98) |
| Bacterial Infection | 2.94 (2.74, 3.14) | 2.63 (2.45, 2.82) |
| **MSOF** |  |  |
| CMV Coincident | 9.47 (8.09, 11.09) | 10.13 (8.64, 11.90) |
| Cytomegalovirus Only | 4.68 (2.86, 7.65) | 4.79 (2.89, 7.92) |
| HSV Coincident | 8.19 (7.20, 9.31) | 7.85 (6.72, 9.17) |
| HSV | 2.51 (1.79, 3.52) | 2.78 (2.04, 3.78) |
| Influenza Coincident | 5.26 (3.28, 8.45) | 5.17 (3.38, 7.91) |
| Influenza | 1.40 (0.38, 5.18) | 1.52 (0.50, 4.63) |
| Bacterial Infection | 5.94 (5.82, 6.07) | 5.14 (5.00, 5.28) |
| **Sepsis** |  |  |
| Coronavirus Coincident | 41.28 (15.48, 110.1) | 65.54 (22.2, 193.3) |
| CMV Coincident | 61.92 (52.66, 72.82) | 67.52 (54.00, 84.44) |
| HSV Coincident | 60.6 (54.2, 67.8) | 62.4 (52.0, 75.0) |
| HSV | 6.07 (3.34, 11.04) | 6.59 (3.42, 12.69) |
| Influenza Coincident | 48.2 (34.3, 67.7) | 45.1 (28.1, 72.6) |
| Influenza | 4.59 (0.68, 30.8) | 4.33 (0.60, 31.3) |
| Bacterial Infection | 51.6 (49.5, 53.8) | 48.8 (46.7, 51.1) |
| **Septic Shock** |  |  |
| CMV Coincident | 200.1 (112.5, 355.7) | 218.7 (128.1, 373.4) |
| HSV Coincident | 308.9 (228.0, 418.3) | 309.2 (211.6, 452.0) |
| HSV | 30.60 (11.53, 81.22) | 33.71 (11.78, 96.41) |
| Influenza Coincident | 130.0 (44.68, 378.5) | 74.6 (23.7, 234.5) |
| Bacterial Infection | 193.3 (166.7, 224.1) | 176.3 (151.8, 204.7) |

*Adjusted for age, gender, race, and hospital cluster

^+^Coincident denotes the presence of both bacterial and viral infections
